# Ethnomycological study in the Kilum-Ijim mountain forest, Northwest Region, Cameroon

**DOI:** 10.1186/s13002-018-0225-8

**Published:** 2018-04-02

**Authors:** N. A. Teke, T. R. Kinge, E. Bechem, T. M. Nji, L. M. Ndam, A. M. Mih

**Affiliations:** 1 Department of Botany and Plant Physiology, Faculty of Science, University of Buea, P.O. Box 63, Buea, South West Region Cameroon; 2 Department of Biological Sciences, Faculty of Science, The University of Bamenda, P.O.Box 39, Bamenda, North West Region Cameroon; 3 Department of Sociology and Anthropology, Faculty of Science, University of Buea, P.O.Box 63, Buea, South West Region Cameroon; 4 Tokyo University of Agriculture and Technology, 3-5-8 Sawai-Cho, Fuchu, Tokyo 183-8509 Japan

**Keywords:** Cameroon, Ethnomycology, Medicinal mushrooms, Traditional knowledge

## Abstract

**Background:**

Majority of the people in rural areas depend on traditional fungi-based medicines to combat different illnesses. This ethnomycological survey was undertaken to document the traditional knowledge of mushrooms among the communities in the Kilum-Ijim mountain forest reserve. Although macrofungi are exploited for food and medicine, their ethnomycological knowledge has not been documented in this ecosystem*.*

**Methods:**

A field study was carried out between 2014 and 2015; 14 mushrooms used by the local communities were collected and identified using the polymorphism of the ribosomal ITS1, 5.8S, and ITS2 regions. Semi-structured questionnaires, focus group discussions, and pictorial method were used to collect information on edibility, local names, indigenous knowledge, and the role of macrofungi in ten communities.

**Results:**

Ethnomycological findings revealed that mushrooms were used as food and medicine, while the non-edible species were regarded as food from Satan. Eight species, *Polyporus tenuiculus*, *Termitomyces striatu*s, *Termitomyces microcarpus Auricularia polytricha, Laetiporus sulphureus, Termitomyces sp.1*, *Termitomyces sp.2*, and *Polyporus dictyopus*, were reported as edible and *Auricularia polytricha*, *Daldinia concentrica*, *Ganoderma applanatum*, *Lentinus squarrosulus*, *Polyporus dictyopus*, *Termitomyces microcarpus*, *Trametes versicolor*, *Vascellum pretense* and *Xylaria* sp., were used as medicine in traditional health care. Local names were found to be a very important factor in distinguishing between edible, medicinal, and poisonous mushrooms. Edible mushrooms are called “awo’oh” in Belo and “Kiwoh” in Oku. Poisonous mushrooms were commonly referred to as “awo’oh Satan” in Belo and “Kiwohfiyini” in Oku. Mushrooms were highly valued as a source of protein and as a substitute for meat in their diets. It is worth noting that *Polyporus dictyopus* was reported here for the first time in literature as an edible mushroom species.

**Conclusion:**

Local knowledge of medicinal mushrooms in the treatment of different illness still exists in all ten villages surveyed. Elderly men and women appear to play an important role in primary health care services in these communities. This survey underscores the need to preserve and document traditional knowledge of the different medicinal mushrooms used in treating different illnesses and for more future scientific research on the mushrooms to determine their efficacy and their safety.

## Background

Fungi are living organisms that are distantly related to plants, and more closely related to animals, but rather different from either of those groups [1]. Fungi represent the greatest eukaryotic diversity on earth and have been for a long time the primary decomposers of lignocellulolytic substrates [2] and the main keepers of great carbon storages in soil and dead organic material [3]. They are key functional components of forest ecosystems [4] and have received less attention than animals and plants though ubiquitous and are highly diverse in nature [5]. Macrofungi inhabit all terrestrial ecosystems with most of them being saprobes or mycorrhizal symbionts, though some are pathogens of plants or fungi [6]. They are extremely common in soil, dead organic matters, nectar, and sap flows. They appear in various morphotypes forming macroscopic fruiting bodies such as gilled fungi, jelly fungi, coral fungi, stinkhorns bracket fungi, puffballs, and bird’s nest fungi [7, 8].

The best known examples of macrofungi are the mushrooms. They have a cap and a stalk and are frequently seen in fields and forests. Under the term “useful mushroom” [9], all the mushrooms which are used by man for some economic importance are considered. Most are simply inedible but there are notable examples that can be eaten. The number of poisonous species is relatively small while those that are fatal belong to a tiny minority. Fungi poisoning and toxic fungi have been investigated in few tropical African countries [10]. At least 3000 species have gastronomic uses and 100 or more have promising clinical activity against cancer and other chronic diseases. The FAO promotes sustainable use of macrofungi for forest management, biodiversity conservation, and their long term effect on income generation and food security [11].

Macrofungi have a long association with humankind and provide profound biological and economic impact. Many wild mushrooms are becoming a ready source of cash for many low-income human beings. From ancient times, wild mushrooms have been consumed by man as delicacy, probably for their taste and pleasing flavor [12]. They have rich nutritional value with high content of proteins, vitamins, minerals, fibers, trace elements, and low/no calories and cholesterol [13]. Many of them have been used in folk medicine for thousands of years. Some of them are nutraceuticals (natural food having potential value in maintaining good health and boosting the immune system of the human body) while others can produce potent nutraceuticals (compounds that have medicinal and nutritional attributes and are consumed as medicines in the form of capsules or tablets but not as food) [14]. Mushrooms are known to be rich sources of various bioactive substances like antibacterial, antifungal, antiviral, antiparasitic, antioxidant, antiinflammatory, antiproli-ferative, anticancer, antitumour, cytotoxic, anti-HIV, hypo-cholesterolemic, antidiabetic, anticoagulant, hepato-protective compounds, among others [15, 16]. Out of approximately 14,000 known species, 2000 are safe for human consumption and about 650 of these possess medicinal properties [17].

In Africa, the knowledge of mushrooms as food, medicine, source of income, and small-scale businesses, and the sociological impacts (myth, culture, and spirituality) are apparently threatened due to slow ethnomycology research drive [18]. Ethnomycological literatures on West Africa are scanty, random, limited in scope, and fraught with taxonomic inconsistencies [18]. In Cameroon and the Kilum-Ijim community in particular, with the current migration, intermarriages and cultural blending; there is also risk of loss of indigenous knowledge of macrofungi. Many mycologists agree that there are more fungi in the world than reported, especially in the tropics and sub-Saharan Africa, and this has resulted in the inconsistency associated with global fungi estimate as reported by [19]. Although there are studies of macrofungi in the domains of systematic, ecology, conservation, ethnomycological surveys, nutritional studies, and cultivation in Cameroon, just about 5% of the total tropical forest zone of 394.700km^2^ has been studied [20].

Cameroon is made up of more than 250 tribes or ethnic groups, each with specific ethnopharmacopoeia on the uses, and behaviors related to various groups of living organisms, such as fungi. Despite this large number of tribes, ethnomycological knowledge is still insufficiently documented within the various tribes. Nevertheless, authors such as [21] and [22] reported the use of 22 and 15 species of edible and medicinal mushrooms, respectively, in the Northwest and Southwest Regions of Cameroon. [23] mentioned the use of some mushrooms as food or medicine around the Mbalmayo Reserve Forest in the Center Region, whereas [24] revealed the common uses of Termitomyces species in the Center and West Regions. [25] investigated the knowledge and utilization of edible mushrooms by the Bantu and Bagyeli (pigmy) populations of the rain forest in the South Region of Cameroon and found that more than 35 species of mushrooms were used as food. In the Kilum-Ijim forest, conservation efforts have been geared towards birds, mammals, and vascular plants with no attention to macrofungi. This is despite the fact that macrofungi do not only play serious socio-economic role in traditional Africa [18], but are principal decomposers and play pivotal roles in the ecological balance [26, 27], in the ecosystem. There is therefore the risk of some fungi going extinct before they are identified.

Considering that the ethnomycological knowledge of various tribes could serve as a tool to assess the fungal diversity in a country, this work is a contribution to the documentation of the edible and medicinal mushroom diversity in Cameroon, particularly of the communities around Kilium-Ijim forest. There is therefore the need for ethnomycological knowledge documentation in the communities around the forest in order to assess species availability as a prelude to conservation and ecological sustainability.

## Methods

### Study area

**Fig. 1:**
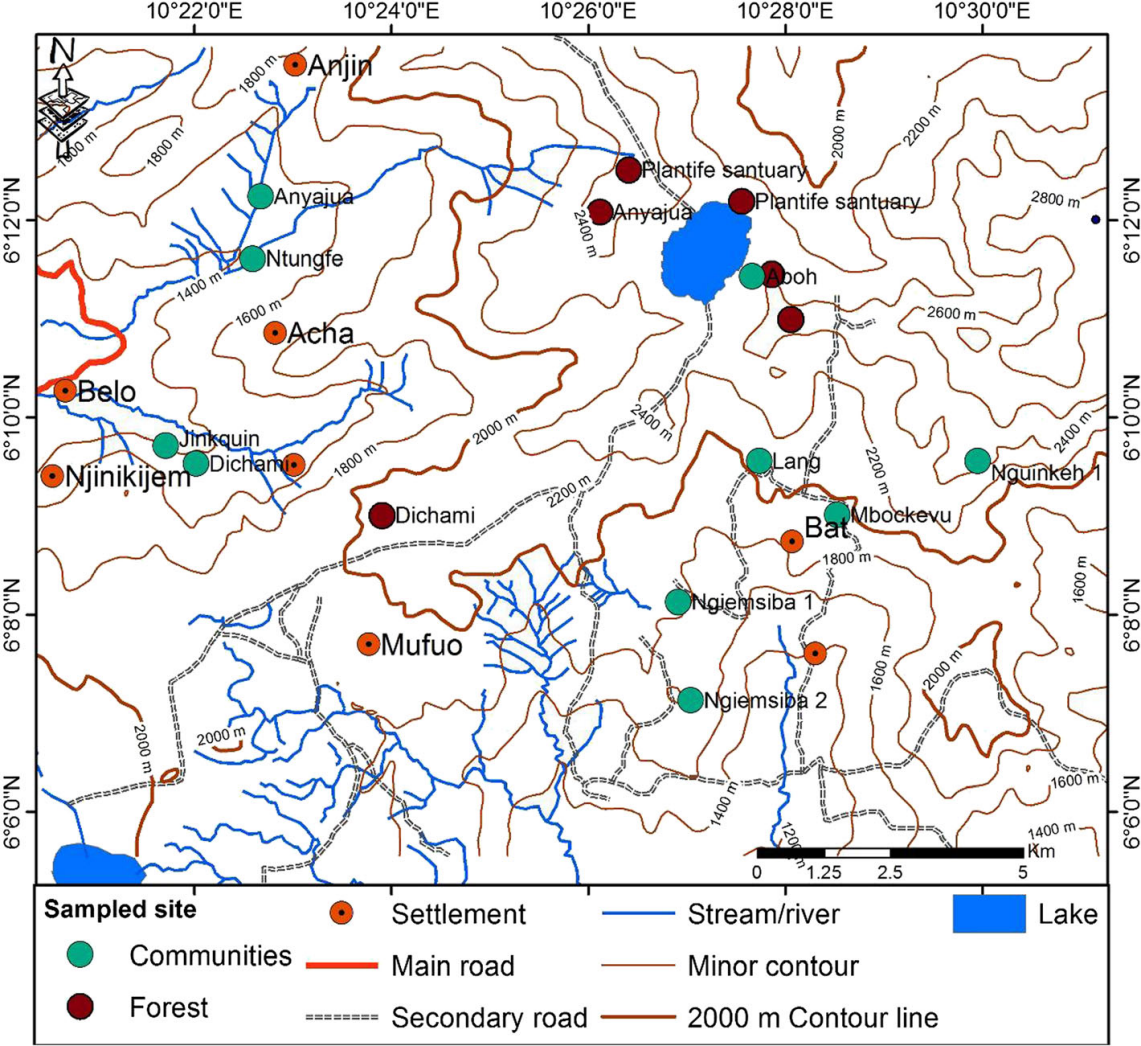
View of the Kilum-Ijim Mountain Forest, Northwest Region, Cameroon

The Kilum Mountain Range and the Ijim ridge are situated in the Northwest region of Cameroon commonly called the Bamenda Highlands. The Kilum-Ijim forest is located between Latitude 6° 07′ N and 6° 17′ N and Longitude 10° 20′ E and 10° 35′ E covering an area of about 20,000 ha (Fig. 1). It is found on Mount Oku with Lake Oku lying in a crater in its center, symbolized by the Lake Oku clawed endemic frog, *Xenopus longipes*. At 3011 m, Mount Oku is the second highest mountain in West Africa, after Mount Cameroon [28].

The forest within this area borders three districts: Oku, Kom, and Nso. Part of the forest that borders Oku is called Kilum, and the rest that borders Kom and Nso is called Ijim—hence, jointly the Kilum-Ijim forest. The Kilum-Ijim forest has been demarcated into 18 community forests to enable better management of the forest resources by the communities surrounding the forest [29]. The climate of the Kilum Mountain is very humid with mist almost throughout the year and very high presence of fog [28].

The precipitation is unimodal [30]. The dry season begins from November to mid-March and the rainy season starts from mid-March to the month of October [30]. The total annual rainfall varies from 1800 to 3000 mm annually, with an average temperature range from 22 °C at 1800 m altitude to 16 °C in the higher altitude areas. The topography of the area is hilly and constitutes a chain of mountains that culminate on mount Oku. The geological landscape found here are mainly of Basalts, trachytes, rhyolites, gneiss, and granite origin.

The soils of this setting are humified ferralitic soils with a high organic matter content and are well drained,and of good permeability [28]. The montane forest has a unique ecosystem that provides a favorable milieu for the habitation of many endemic plant and bird species [30]. The area is one of the most densely populated parts of Cameroon. It is estimated that close to 300,000 people live within a day’s walk of the forest [31]. The majority of the area enclosed by the Kilum-Ijim boundary is at an altitude of over 2000 m. The vegetation at this altitude and above consists mainly of montane forest mixed with montane grassland and subalpine communities. Below this level, most of the submontane forest has already disappeared due to clearance for agriculture.

### Macrofungi collection and identification survey

**Fig. 2:**
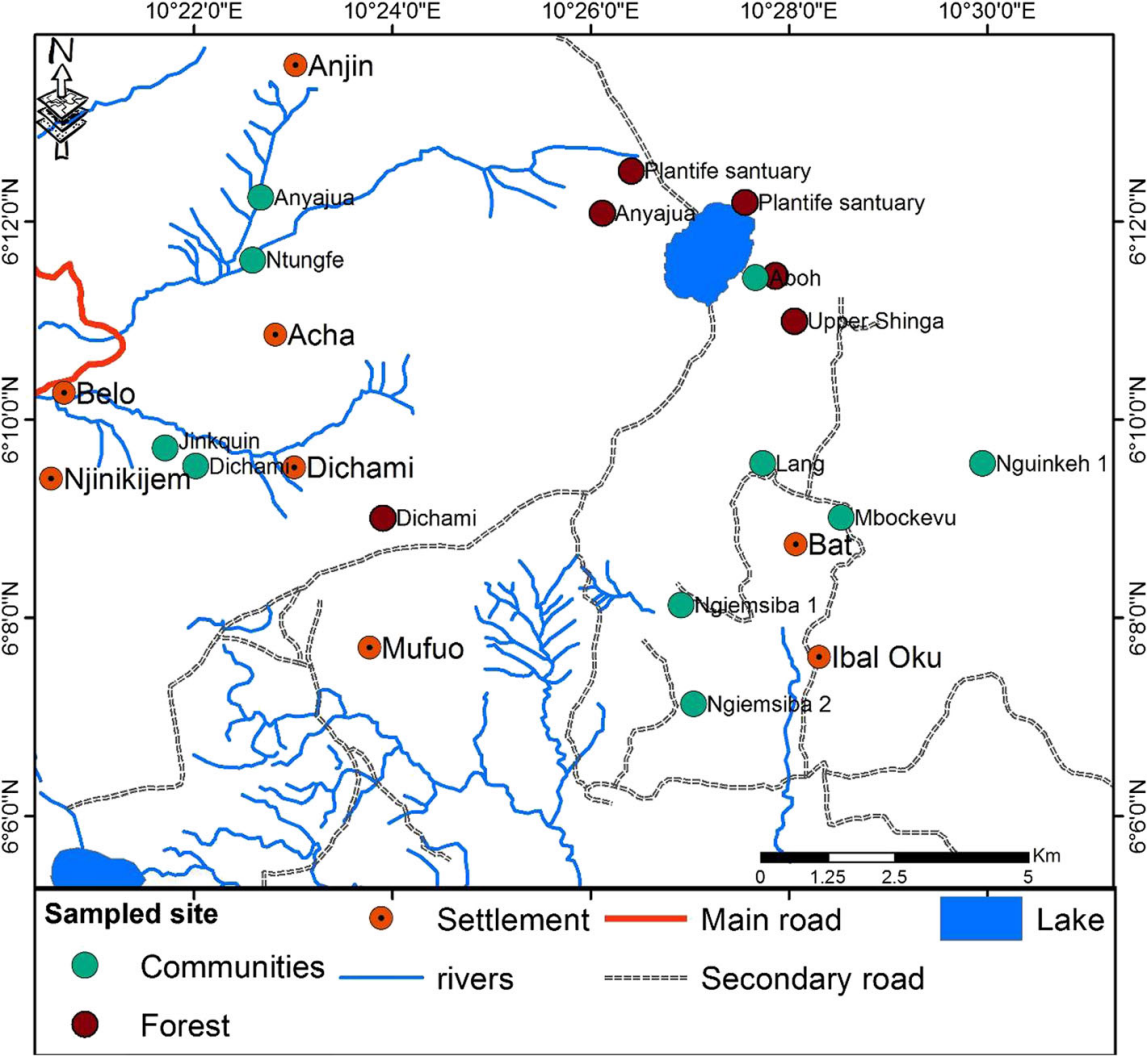
Ethnomycological study sites of macrofungi in Kilum-Ijim Mountain Forest, Northwest Region, Cameroon

Before entering into the Kilum-Ijim forest, visitations were made to all the fondoms and administrative authorities within the area to sought traditional and administrative clearance to forest usage. Five community forests (Fig. 2) were selected for sample collection based on accessibility after a reconnaissance survey was carried out in the area.

Macrofungi identification and collection were done by opportunistic sampling with the assistance of informants in the different villages.

For each macrofungus collected, the fungus was labeled, the growth substrate recorded and the morphological characters assessed. These attributes included cap shape, stipe length, size, and color. This information was used to place the fungus on different morphotypes according to [6]. Photographs of labeled samples were recorded since some features can change during preservation. Macrofungal samples were dug off the soil or cut off with a knife where they were found on wood substrata. The samples were wrapped with their tags using an aluminum foil and put in ziplock bags for drying. Drying was done in a locally designed open air oven at 45–55 °C for 72 h for fleshy thick samples. The dried samples were carefully wrapped in absorbant papers and preserved over blue silica gel in ziplock bags for later identification while duplicates were deposited in the University of Buea hebarium. Molecular identification was carried out in the Biosciences eastern and central Africa (BecA) laboratory Nairobi, Kenya, as reported by [32] in which total DNA was extracted from powdered samples using the DNeasy® Plant Mini Kit (QIAGENGroup) as per the manufacturer’s protocol with minor modifications. DNA concentration and purity were determined by NanoDrop spectrophotometer (Thermo Scientific NanoDrop 2000) at absorbance (A260/280). Amplification of the ITS1, 5.8S and ITS2 regions for assessing ITS length variation was done using primer ITS1F (5′-TCCGTAGGTGAACCTGCGG-3′) [33] and ITS4 (5′TCCTCCGCTTATTGATATGC-3′) [34].

PCR amplification was done using AccuPower® Taq PCR premix (Bioneer, www.bioneer.com) in a 20 μL reaction volume containing 50 ng template DNA, 0.18 μM of each primer, and 15.1 μL Milli-Q water was added. The thermocycler settings were as follows: denaturation at 95 °C for 3 min; 35 cycles of denaturing at 94 °C for 30 s, annealing at 55 °C for 30 s, and extension at 72 °C for 1 min; and a final extension at 72 °C for 10 min. 2 μL of each PCR product were electrophoretically separated on a 2% agarose gel prepared in 0.5X TAE to check the purity of the PCR product. The gel was run at 7 V/cm for 45 mins. DNA staining was done with 0.025X GelRed and photographed under UV exposure. The PCR products were then purified using QIAGEN® purification kit following the manufacturer’s instructions. The purified PCR products were sent to Macrogen (Netherlands) for Sanger sequencing. Related gene sequences for each of the macrofungal specimens were obtained from NCBI GenBank using UNITE ITS Database and then automatically aligned using CLC Main Workbench. Multiple sequence alignments were then performed in MEGA6 [35] to allow maximum sequence similarity.

### Ethnomycological survey

The ethnomycological survey sought to evaluate the recognition, role, and conservation status of macrofungi as viewed by the inhabitants of the Kilum-Ijim region. The survey was carried out in 10 communities including; Aboh, Ntungfe, Anyajua, Dichami, Jinkquin, Mbockevu, Nguinkeh 1, Lang Ngiemsiba 1, and Ngiemsiba 2. These communities were selected based on their proximity to the forest. Instruments used to collect information from community members included semi-structured questionnaires, focus group discussions (FGD), and pictorial presentation (show-and-tell method). The distribution of the different instruments is summarized on Table 1.

**Table 1:** Sample size distribution for different instruments used in ethnomycological survey in Kilum-Ijim Mountain Forest, Northwest Region, Cameroon

Community	No. of respondents	No. of FGD	No. of pictorial presentations
Aboh	50	2	2
Ntungfe	50	2	2
Anyajua	50	2	2
Dichami	50	2	2
Jinkquin	50	2	2
Mbockevu	50	2	2
Nguinkeh 1	50	2	2
Lang	50	2	2
Ngiemsiba 1	50	2	2
Ngiemsiba 2	50	2	2
Total	500	20	20

A total of 500 questionnaires were randomly distributed in the different communities. Information was sent to the community members through their village heads before each visit, and prior to each interview, the informant’s consent was sought. The questionnaire was constructed to obtain vital information including local names and their meanings, substrates of mushrooms and fruiting periods, uses of mushrooms (food or medicine), methods of preparation, and criteria for differentiating toxic and edible mushrooms. Also included were questions on sociodemographic information including age, gender, and occupation. Focus group discussions (FGD) consisted mainly of traditional practitioners and adult men and women above 30 residing in that community who are believed to have good knowledge on mushrooms. Each focus group discussion was made up of 8–12 persons comprising of at least three traditional practitioners. Information from focus group discussions were recorded using microtapes and transcribing done after. Pictorial presentations were done to compliment and authenticate information obtained from questionnaires and FGD. The pictorial charts comprised 200 species of mushrooms collected from diversity survey. The aim was for the community members to identify and give their local names and uses. Descriptive statistics were used to analyze the ethnomycological data collected.

## Results

### Morphological and molecular identification of macrofungi species

**Fig. 3:**
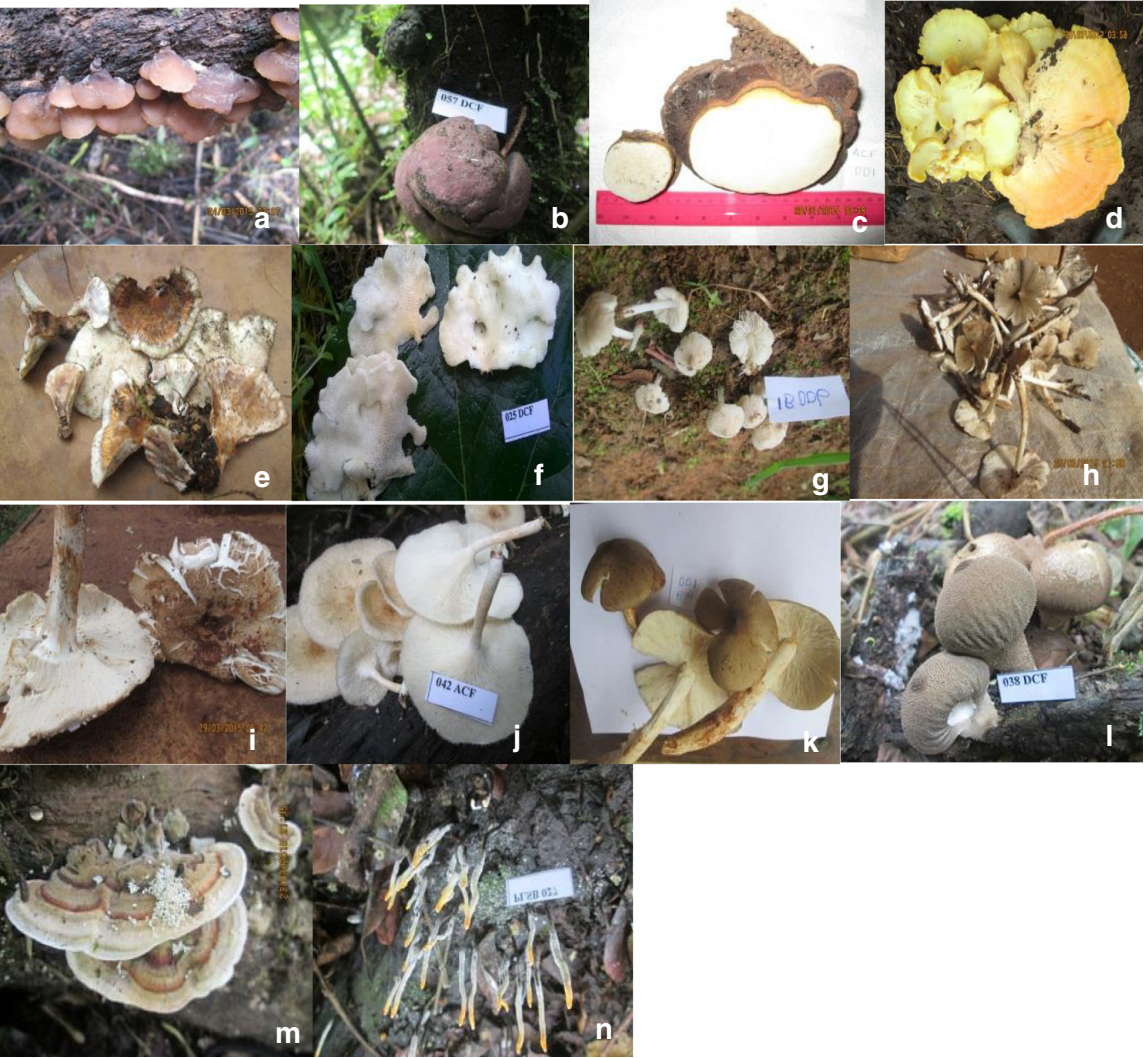
Edible and medicinal mushrooms known and utilized by inhabitants in Kilum-Ijim Mountain Forest, Northwest Region, Cameroon. **a** *Auricularia polytricha*, **b** *Daldinia concentrica*, **c** *Ganoderma applanatum*, **d** *Laetiporus sulphureus*, **e** *Polyporus dictyopus*, **f** *Polyporus tenuiculus*, **g** *Termitomyces microcarpus*, **h** *Termitomyces* sp. 1, **i** *Termitomyces* sp. 2, **j** *Lentinus squarrosulus*, **k** *Termitomyces straitus*, **l** *Trametes versicolor*, **m** *Vascellum pretense*, **n** *Xylaria sp.*

Based on morphological and molecular identification, the species used in ethnomycology were identified into 14 species: *Auricularia polytricha*, *Daldinia concentrica*, *Ganoderma applanatum*, *Laetiporus sulphurous*, *Lentinus squarrosulus*, *Polyporus dictyopus*, *Polyporus tenuiculus*, *Termitomyces microcarpus*, *Termitomyces sp.1*, *Termitomyces sp.2*, *Termitomyces striatus*, *Trametes versicolor*, *Vascellum pretense* and *Xylaria* sp. (Fig. 3).

### Demographic information of the population

Results from questionnaires revealed that of the 500 respondents 41% were between the ages of 36 and 56 years, 31% between 15 and 35 years, and 28% between 57 and 71 years. Females recorded 67.4% of the respondents. The occupation of the inhabitants was mainly farming (Table 2).

**Table 2:** Demographic profiles of the surveyed communities in Kilum-Ijim forest, North West Cameroon

Community	No. of respondents	Age group		Gender			Civil status		Occupation	
		15–35	36–56	57–71	Male	Female	Single	Married	Farming	Others
Aboh	50	14	20	16	18	32	16	14	46	04
Ntungfe	50	16	22	12	09	41	23	27	39	11
Anyajua	50	14	21	15	20	30	17	33	44	06
Dichami	50	15	22	13	16	34	28	22	41	09
Jinkquin	50	16	18	16	14	36	18	32	42	08
Mbockevu	50	16	22	12	19	31	22	28	45	05
Nguinkeh 1	50	14	20	16	17	33	13	37	43	07
Lang	50	17	20	13	23	27	15	35	41	09
Ngiemsiba 1	50	18	21	11	15	35	19	31	43	07
Ngiemsiba 2	50	15	19	18	12	38	14	36	46	04
Total	500	155	205	140	163	337	185	315	430	70

#### Ethnomycological knowledge of mushrooms

Information from this survey revealed that the inhabitants of Kilum-Ijim have a high mycophilic culture for mushrooms. All respondents (100%) reported mushrooms as food, while 30% of the respondents added that mushrooms were also used to treat various ailments. Mushrooms were considered as a substitute for meat and highly preferred for their nutritive value, flavor, and taste especially during fruiting seasons.

Mushrooms are classified into two categories: edible/medicinal and poisonous. The edible mushrooms are called “awo’oh” in Belo and “Kiwoh” in Oku. Inedible mushrooms were commonly referred to as “awo’oh Satan” in Belo and “Kiwoh fiyini” in Oku.

Fourteen species were recorded as edible/medicinal by the Kilum-Ijim people. Vernacular names of the edible mushrooms were usually associated with the features of the mushroom or the substrates on which they grew (Table 3).

**Table 3:** Inventory of edible and medicinal mushrooms collected from the Kilum-Ijim forest North West Cameroon

Sample collection no.	Species	Vernacular name	Meaning	Habitat	Period of collection	Use
Agaricaceae						
PLSB038	*Vascellum pretense* (Pers.) Kreise	Not known	Not known	Soil	March-Oct	Medicine
Auriculariaceae						
DCF058	*Auricularia polytricha* (Mont.) Sacc.	Vutungleh	Ear shaped	Decomposing wood	March-June	Food/medicine
Ganodermataceae						
ACF001	*Ganoderma applanatum* (Pers.) Pat.	Awo’oh fuka	Mushroom on wood	Decomposing wood	All year round	Medicine
Lyophyllaceae						
IBAL001	*Termitomyces microcarpus* (Berk.& Broome) R. Heim	Futiatih/Mutotos	Tiny mushrooms	Termite hills	March-April	Food/medicine
IBAL002	*Termitomyces sp.1* R.Heim	Awo’oh/kiwo’oh	Mushroom on wood	Termite hills	March-April	Food
IBAL003	*Termitomyces sp.2* R.Heim	Awo’oh/kiwo’oh	Mushroom on wood	Termite hills	March-April	Food
BAT001	*Termitomyces striatus* (Beeli) R.Heim	Awo’oh/kiwo’oh	Mushroom on wood	Termite hills	March-April	Food
Polyporaceae						
USCF003	*Laetiporus sulphureus* (Bull.) Murrill	Allem/kilim	“Meatlike”	Decomposing wood	March-June	Food
ACF006	*Lentinus squarrosulus* Mont. 1842	Ntaghagheus	Tough skin	Decomposing wood	March-June	Food/medicine
DCF025	*Polyporus dictyopus* Mont. 1835	Ntaghagheus	Tough skin	Decomposing wood	March-Oct	Food/medicine
DCF15	*Polyporus tenuiculus* (Beauv.) Fr.	Ntaghagheus	Tough skin	Decomposing wood	March-Oct	Food
PLSO004	*Trametes versicolor* (L.) Lloyd	Awo’oh fuka	Mushroom on wood	Decomposing wood	March-Oct	Medicine
Xylariaceae						
DCF057	*Daldinia concentrica* (Bolton) Cesati & de Notaris	Awo’oh fuka	Mushroom on wood	Standing wood	March-Oct	Medicine
PLSB27	*Xylaria* sp. Hill ex Schrank	Awo’oh fuka	Mushroom on wood	Decomposing wood	All year round	Medicine

#### Criteria for determining edibility

**Fig. 4:**
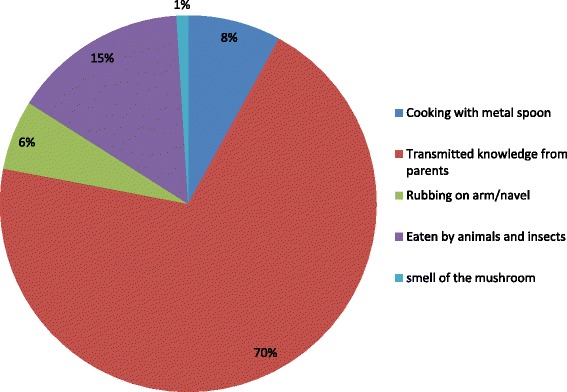
Methods of determining edibility of mushrooms in Kilum-Ijim Mountain Forest, Northwest Region, Cameroon

The people of Kilum-Ijim have different ways by which they determined edibility of mushroom species (Fig. 4). It was reported that the knowledge of edibility was transmitted from parents to children or by observing if the mushroom was eaten by insects and other animals. Other methods included rubbing on parts of the body particularly the arm or navel to test for skin irritation. Skin irritation indicated that the mushroom species is not edible. Another method was by cooking with metal spoons. If the spoon turns black, it indicated that the species is not edible.

#### Species preference ranking

Results from preferences rankings of the different mushroom species recorded as food revealed that the soil-inhabiting species (*Termitomyces* sp. 1, *Termitomyces microcarpus*, *Termitomyces* sp. 2 and *Termitomyces striatus*) were most preferred than the wood-inhabiting species (*Auricularia polytricha*, *Laetiporus sulphureus*, *Polyporus dictyopus*, *and Polyporus tenuiculus*) which received the least rating in consumer preference (Table 4).

**Table 4:** Preference ranking of eight edible mushroom species consumed in in Kilum-Ijim forest, North West Region, Cameroon

Mushroom species	Respondents									Score	Rank
	1	2	3	4	5	6	7	8	9		
*Termitomyces striatus*	7	5	4	6	5	8	4	6	4	49	4th
*Termitomyces* sp.1	7	6	7	8	7	5	6	9	9	64	1st
*Polyporus tenuiculus*	5	5	6	4	3	5	2	5	4	39	8th
*Termitomyces microcarpus*	9	8	6	5	8	4	7	8	5	60	2nd
*Auricularia polytricha*	8	7	5	3	8	3	4	6	3	47	5th
*Laetiporus sulphureus*	9	6	5	3	4	5	2	7	3	44	6th
*Polyporus dictyopus*	6	5	7	4	3	6	2	5	4	42	7th
*Termitomyces* sp. 2	6	4	8	7	5	8	7	4	6	55	3rd

#### Preparation and mode of consumption of wild mushrooms

**Fig. 5:**
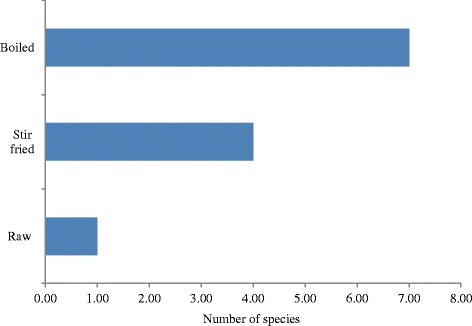
Mode of consumption of edible mushroom species in Kilum-Ijim Mountain Forest, Northwest Region, Cameroon

Analysis of the mode of consumption of the edible mushroom species revealed that out of the 12 mushroom species considered as edible, 7 were boiled, 4 stir-fried, and 1 species eaten raw (Fig. 5). Species of *Termitomyces*, *Auricularia*, *and Laetiporus* were regarded as “meat” and were usually added to soups after boiling or fried with vegetables. They believed that mushrooms are tastier than meat and are also rich in proteins. Mushrooms are usually prepared in groundnut soups, “achu” and “okro”. Young fruiting bodies of *Laetiporus sulphureus* are usually eaten raw as snacks. Our study revealed that all edible species were prepared fresh after harvesting except *Termitomyces* species that were usually dried in the sun or smoked and preserved in airtight containers for future use.

#### Medicinal mushrooms utilized in the Kilum-Ijim region

The study revealed that nine mushroom species were used to treat various ailments by the inhabitants of Kilum-Ijim. The mode of administration was usually oral, and they were either drunk as concoctions, decoctions, or in soups (Table 5).

**Table 5:** Medicinal mushrooms known and used by the population in in Kilum-Ijim forest, North West Region, Cameroon

Species	Ailment treated	Method of preparation	Method of administration
*Auricularia polytricha*	Nausea in pregnant women	Cooked with soup	Oral
*Daldinia concentrica*	Hypertension	Sliced and boiled with other herbs	Oral
*Ganoderma applanatum*	Builds up immune system	Boiled in water	Oral
*Lentinus squarrosulus*	System cleansing	Cooked with soup	Oral
*Polyporus dictyopus*	Stomach aches and head aches	Boiled with other herbs	Oral
*Termitomyces microcarpus*	Bone strengthening in children and Fever	Cooked with soup, boiled in water and drunk	Oral
*Trametes versicolor*	Strengthens immune system	Boiled in water and drunk	Oral
*Vascellum pretense*	Fever	Mixed with other herbs and boiled	Oral
*Xylaria* sp.	Hypertension, fever	Added to herbal preparations	Oral

#### Indigenous beliefs on mushroom appearance and collection

The people of Kilum-Ijim had several indigenous beliefs on mushroom appearances and collection. They believed that during the rainy season, lightning strikes the ground to stimulate the appearances of mushrooms. To them, the appearance of mushrooms was a free gift and miracle from God and a sign of good luck to whoever sees them first. Mushrooms were not harvested all when discovered, and after harvesting, the remnants were mixed with salt and sprinkled on the place of harvest to enable a richer harvest in the future. Another myth was that during harvesting, the harvested mushrooms must be shared to any passerby who sees you as a sign of gratitude to God. Failure to share resulted in curses on your family or decay of corpse before burial.

#### Economic values of edible mushrooms

*Termitomyces* species had high economic value as compared to the other edible mushroom species consumed by the inhabitants of the Kilum-Ijim region. During the fruiting periods of March to May, these species were harvested and sold. Income generated from sales was used to improve livelihoods of these inhabitants. Results revealed that these mushrooms were sold in sizes ranging from handful heaps to 20 l basketful at prices ranging from 100FCFA to 10000FCFA (10cents–20USD) respectively. This income enabled them to also increase their livelihoods and living standards.

#### Threats and conservation of wild mushrooms

Anthropogenic activities such as deforestation for farmlands, settlement expansion and grazing sites, and fire outbreaks, by the local communities around the Kilum-Ijim forest, were causing great threats to the macrofungi population in this forest. About 80% of the elderly respondents reported that a lot of macrofungi have disappeared resulting in the loss of ethnomycological knowledge. Results revealed that though there are laws and policies on biodiversity conservation in Cameroon, these laws were not being implemented in the Kilum-Ijim forest especially with regard to fungi conservation. This however exposes the fungi to risk of extinction.

## Discussion

### Traditional knowledge

Macrofungi play essential roles in maintaining forest ecosystems and biodiversity [35, 36].This study set out to evaluate the ethnomycology in the Kilum-Ijim forest. Based on ethnomycological knowledge, our findings revealed that the inhabitants of Kilum-Ijim had a very good knowledge on mushrooms. Mushrooms were used as food and medicine and as source of income depending on seasonal availability. The collection of mushrooms by the people of Kilum-Ijim was usually combined with other activities like farming, honey harvesting, or hunting when they visited the forest. When collected in excess, the mushrooms were sold in local markets to raise income for livelihood.

The Kilum-Ijim people had different ways of determining edibility of mushrooms. The knowledge on edibility was largely transmitted from parents to offspring or based on color and smell or identified as being eaten by animals or insects. Similar reports on this transmitted knowledge have been reported by [25] and [22] in the South and Southwest region of Cameroon respectively. [37] reported that color and smell of mushroom was also used as a criterion to determine edibility by the inhabitants of Tlayacapan, Morelos in Mexico. Results also revealed that mushrooms which caused blackening of silver spoons upon boiling were considered harmful by the Kilum-Ijim people. Similar findings have been reported by [38] who studied mycophagy in Central and Eastern Europe.

Traditional names of commonly consumed mushrooms were obtained with nine species belonging to five genera identified. Edible mushrooms are generally referred to as “awo’oh” by the people of Belo and “Kiwo’oh” by the Oku people. Macrofungi with bad odors especially species of *Phallus* and *Clathrus* were referred to as “awo’oh Satan” in Kom and “kiwo’oh fiyini” in Oku, meaning “mushrooms from the evil one”.

The Belo people attribute the names of the mushrooms based on the substrate on where they occur. The name “awo’oh fuka” is referred to edible species on wood and “awo’oh diya” to edible ground species. This is similar to the findings of [25] in the South region of Cameroon where the name of the fungus was attributed to the substrate on which it occurs.

The inhabitants of Kilum-Ijim had several indigenous beliefs on mushroom collection and utilization. There was a wide belief that mushrooms appearance were as a results of lightening and miracles of God. These beliefs have been corroborated by [23] and [39] on their studies on ethnomycology in the Mbalmayo forest reserve, Cameroon and Igala land, Nigeria, respectively. A very interesting study was the cultural belief attached to some edible species by the Kilum-Ijim people. For example, the common belief concerning *Termitomyces striatus* was that after harvesting, the remnants are mixed with salt and sprinkled on the place of harvest to enable a richer harvest in the future. Also, during harvesting, the harvested mushrooms must be shared to any passerby, as a sign of gratitude to God. Failure to share resulted in curses on your family or decaying of corpse before burial. Strange beliefs with regards to *Termitomyces* species have also been reported by [39] by the people of Igala land in Nigeria.

The study revealed that only species of the genus *Termitomyces* were sold in the local markets during fruiting seasons. This implies that this genus contains species which are highly preferred for their taste, flavor and nutritional benefits. Market values of mushrooms ranged from 100FCFA (10 cents) for a handful heap to 10000FCFA (20 USD) for a basketful size. Income generated from these sales was used to improve the livelihood of the inhabitants. Several reports have revealed that *Termitomyces* species have high economic value, and their sales greatly contribute in adding the income of many households in local communities [21, 40, 41].

The study showed that mushroom hunting was mostly done by children and women who usually cooked some at home and also sold some on market days to increase household incomes. However, some men reported that they collected mushrooms when they accidentally came across them on their farmlands or in the forest. About 65% of the interviewed population reported that they do not believe that mushrooms could be cultivated. They strongly believe that mushrooms were gifts from God and no one could cultivate them. However, 35% of the respondent had knowledge of mushroom cultivation and were interested to learn the cultivation if they were taught.

About 80% of the elderly respondents reported that a lot of macrofungi have disappeared resulting in the loss of ethnomycological knowledge. They attributed these disappearances to habitat degradation of the ecosystem for farmlands, grazing sites, and settlement expansion. This observation is similar to the findings of [42] among the Igbo people in Nigeria.

### Mushrooms used in the treatment of different illness

Medicinal use of mushrooms among the Kilum-Ijim inhabitants was not as strong as those reported by [43]. The Kilum-Ijim inhabitants used mushrooms mainly as food and rarely for non-food purposes. Only about 30% of the interviewed population had knowledge of the use of mushrooms as medicine. Among the medicinal mushrooms reported by the Kilum-Ijim inhabitants, the genera *Termitomyces*, *Auricularia*, *Ganoderma*, *and Trametes* have also been reported by [22] as useful mushrooms in the treatment of different illnesses by the Bakweri people of the Southwest region of Cameroon. The inhabitants of Kilum-Ijim preferred mushrooms as food, acting as a substitute for meat in their diets due to their taste and flavor. This finding is similar to earlier studies by [44] and [20] who both reported that most people eat mushrooms mostly because of their flavor and taste and as a substitute for meat. There was no difference among the respondents with regards to the names, usage, and perception of mushrooms by the inhabitants of the Belo subdivision and the inhabitants of the Oku subdivision.

Ethnomycology results described *Polyporus dictyopus* as edible for the first time in Cameroon literature. It was observed that some mushrooms considered inedible among the Kilum-Ijim people were reported as edible in other regions of Cameroon by inhabitants who had traveled to these regions. Therefore, there is still great variation in ethnomycological knowledge of mushrooms.

## Conclusions

Macrofungi play important roles in the socio-economic life of the inhabitants of the kilum-Ijim region. Ethnomycological knowledge on indigenous mushroom species in the Kilum-Ijim region is facing risk of extinction due to lack of documented information and the aging away of the older generation. Secondly, a lot of inconsistencies still exist as to which mushrooms are edible or poisonous.

Macrofungi do not only have socio-economic benefits, but also play essential roles in ecosystem functioning. These organisms are minimized in conservation laws and policies. Conservation through the implementation of laws and policies will not only preserve ethnomycological knowledge and extinction of these species, but will also help in the conservation of the ecosystem as a whole.

Macrofungi do not only have socio-economic benefits, but also have important functional roles in ecosystems. Government laws and policies should be implemented to ensure their conservation alongside plants and animals. Ethnomycological knowledge which is inherited over generations is of high cultural value; it is important that more research be done in documenting this information.
